# Essential human and material resources for emergency care in the district hospitals of Burundi

**DOI:** 10.1016/j.afjem.2023.09.005

**Published:** 2023-10-13

**Authors:** Thierry Giriteka, Doña Patricia Bulakali, Carlan Bruce Wendler

**Affiliations:** a Kibuye Hope Hospital, Kibuye, Bukirasazi, Gitega, Burundi; b Hope Africa University, Ngagara II, Bujumbura, Burundi

**Keywords:** District hospital, Emergency care, Essential medications, LMIC, Ultrasound capacity

## Abstract

**Introduction:**

Burundi, like many African nations, faces challenges in providing accessible emergency care. The aim of this study was to assess the type of staff training, accessibility to imaging, and availability of essential equipment in the district hospitals of Burundi in order to inform strategic planning for healthcare delivery.

**Methods:**

In June 2022 an online survey was sent to each district hospital of the country. Complete responses were analysed and, where appropriate, significance determined by chi-square analysis, with *p*<0.05 considered significant.

**Results:**

Forty of 45 district hospitals completed the survey, of which 35 were rural (matching national demographics). The majority of district hospitals (21/40) had ready access to ≥4/5 critical drugs while few (5/40) were equipped with ≥4/5 key material. One quarter had 24/7 physician coverage and X-ray available. Only 3 had continuous access to ultrasound studies despite most district hospitals having ultrasound machines. Trained emergency room staff were almost totally absent from the field, with only 6 nurses, 4 generalists, and 1 specialist reported across 9 sites. Even a single EM-trained staff member was significantly correlated with being better equipped for emergencies (*p*<0.01).

**Conclusion:**

Burundi needs a strategic investment in emergency preparedness and care. Policy initiatives and technology purchases have demonstrated reasonable penetration down to the district hospital level, however, trained personnel are essential to develop sustainable emergency capacity.

## African relevance

▒

• The vast majority of healthcare provision in Africa occurs at or below the level of the district hospital.
• Training of personnel and equipping district hospitals with key material resources can save lives, reduce disability, and prevent tragedy in Africa.

▒

## Introduction

### African developments in emergency medicine

The twin tides of urbanization and industrialization sweeping the fastest-growing continent make urgent the development of robust systems for emergency care in Africa. The number of medical, surgical and traumatic emergencies continue to increase globally and in Africa [1], [2], [3], [4]. Access to timely emergency care in Africa is improving but low income countries still have significant challenges that include resource limitations and lack of training for staff [5], [6], [7], [8]. Though important for planning healthcare delivery systems, data on capacity to manage critically-ill patients (physical resources and healthcare professionals) are often lacking [9], [10], [11].

The World Health Organization (WHO) recommends trained and well-equipped staff for emergencies as elements of preparedness. The WHO and other international non-governmental organizations (NGOs) work hard to provide assistance and strengthen country and community emergency preparedness in order to ensure a timely, efficient and effective response. However, low- and middle-income countries (LMICs) still have an enormous burden compared with higher-income countries [12], [13], [14].

Most sub-Saharan African countries have hospitals close at hand to the population capable of providing emergency medical services, with 71 % of the population living less than 2 h away from these hospitals [15]. In spite of this, many of these hospitals remain inadequately equipped [5], [6], [7], [8]. This is associated with overall mortality rates as high as 15 % in emergency departments in African nations [16].

### Burundian demography & healthcare delivery

Burundi is a central African country and member of the East African Community. According to demographic projections from 2008 (date of last census), in 2022 Burundi had a population of 12.8 M, of which 50.7 % was female and 13.2  % under 5 years old [17]. The healthcare delivery system in Burundi is arranged in four levels which form the chain of referral. The Ministry of Public Health and the Fight against HIV/AIDS, via its three general directorates, supervises the 18 Provincial Health Offices whose mission is to ensure the implementation of the health policy in their area of responsibility [18]. Below the provinces, 47 health districts manage the 1182 health centers and the 45 district hospitals that form the first link in the referral chain [18,19]. These district hospitals transfer patients to the five regional hospitals who then refer the most critical or complicated patients to the seven national referral hospitals. Of the seven tertiary referral hospitals, six are located in Bujumbura, the largest city comprising 6  % of the Burundian population [17,19].

### Medications, material, and training

Since 1977, the World Health Organization has published its Essential Medicines List with an additional list of critical medications for the care of children added from 2007 onwards [20]. These lists contain over 700 medicine and therapeutic equivalents. Among these are many deemed necessary to be stocked in the ED where their timely administration may make the difference between saving a life and losing it. The nearest comparable list of essential medical devices or equipment (herein, “material”) was published in 2021 [21] and during the COVID-19 pandemic, when the WHO released a list of priority medical devices, including oxygen, pulse oximetry, X-ray, and ultrasound machines [22]. Other studies have shown that early access to defibrillation, even by bystanders without medical training, can reduce the morbidity and mortality of cardiac arrhythmias, making automated external defibrillators a logical inclusion in any such lists though their cost-effectiveness in settings with low incidences of vascular heart disease merits careful investigation [23,24]. Regarding training in essential resuscitation and stabilization skills, the nearest equivalent to the half-century old Essential Medicines List is the Basic Emergency Care (BEC) course, released jointly by the WHO, International Committee of the Red Cross and International Federation of Emergency Medicine in 2016. Targeting emergency clinical care providers of all levels, the elements of the BEC are instantly recognizable as the core components of emergency medical, obstetrical, surgical and trauma resuscitation. Its implementation has had positive effects in numerous resource-limited settings, including several nations in sub-Saharan Africa [25], [26], [27], [28]. These results are so positive that it is likely that any formal training in essential emergency care skills and protocols can improve provider confidence and patient care.

Moreover, adequately staffed and equipped emergency departments save lives and reduce morbidity [12]. Training emergency care providers improves outcomes in a wide range of common conditions [29]. To that end, Burundi is making efforts to improve and be prepared to respond to emergencies and natural disasters. It has placed prevention, preparedness and response to emergencies and disasters among the priorities of the national health strategy 2016–2025, as this remains a major challenge with a large gap between the real needs of populations in complex emergency situations and the availability of appropriate multi-sectoral services to prevent or respond to cases of sudden exposure to emergencies and/or natural disasters [30]. At the time of this survey, no studies have assessed the preparedness of the Burundian healthcare delivery system to respond to common medical emergencies at the district hospital level.

The aim of this study was to assess the type of staff training, accessibility to imaging, and availability of essential equipment in the district hospitals of Burundi in order to inform strategic planning for healthcare delivery.

## Methods

### Study design

This cross-sectional study was conducted to assess the preparedness of district hospitals to receive critically ill patients, with regard to the accessibility of essential equipment, imaging facilities and human resources for emergency services.

### Data collection, management, and analysis

We used a multimodal communication model to recruit respondents from every district hospital in Burundi for an online survey in June 2022. The questionnaire was developed by the authors on the basis of WHO recommendations and the operational guide for health district management in Burundi, to assess the accessibility of essential equipment, imaging facilities and human resources to emergency services.

The authors sent a web-based questionnaire (see Appendix) via WhatsApp or text message to medical directors of the District Hospitals. Any healthcare worker from the target hospitals was allowed to complete the survey and hospitals with multiple responses were cross-checked for accuracy and any cases of discordance were followed by an individualised inquiry. Hospitals that did not complete the form during the two-week data collection window were contacted via email and/or phone call with a reminder invitation to complete the survey. No incomplete surveys were received and no protected healthcare information or identifiers were collected.

We assessed the availability in the Emergency Room within 50 m or a 5 min walk of certain essential medications selected from the WHO Essential Drugs list with attempt made to cover a variety of typical conditions for the Burundian context in the fewest number of items so as to improve completion rate and avoid the problems associated with incomplete datasets and small sample sizes [31]. Hospitals with ≥4/5 of these drugs were classified as “supplied,” those with 3/5 as "partially supplied" and those with <3/5 as "not supplied". Similarly, hospitals with ≥4/5 of the emergency equipment included in the study (excluding 24/7 radiology availability) were classified as "equipped", those with 3/5 as "partially equipped" and those with <3/5 categorised as "not equipped".

Responses were correlated to demographic profiles derived from the Burundian national census database to establish societal impacts [32,33]. Where appropriate, tests of statistical significance were calculated using chi-square analysis with p-value <0.05 considered significant.

### Ethical approval

Formal review by the Hope Africa University School of Medicine research ethics committee was not sought as the study met exemption criteria (retrospective and anonymised data collection only, no direct impact on clinical care, no vulnerable population involved). Authorization from the medical directors of the responding District Hospitals was obtained prior to data collection.

## Results

### Personnel

**Fig. 1:**
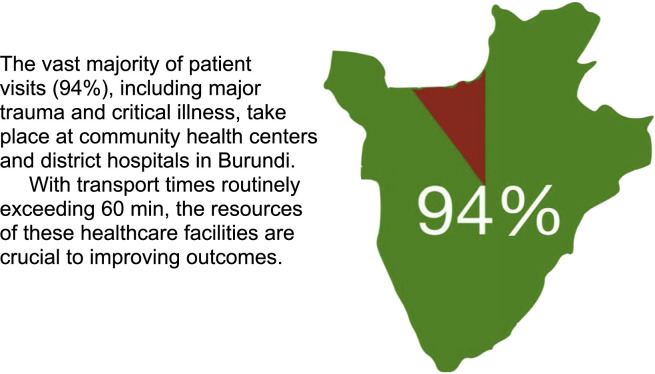
Depiction of care provision in Burundi The vast majority of patient visits (94 %), including major trauma and critical illness, take place at community health centers and district hospitals in Burundi. With transport times routinely exceeding 60 min, the resources of these healthcare facilities are crucial to improving outcomes.

Forty of 45 district hospitals (89  %) provided complete data for the study, representing 90  % of the Burundian population. The majority (35/40, 88  %) of responding hospitals were in rural districts, which matches closely the population distribution of Burundi. Only a quarter of represented ERs claimed 24/7 physician coverage, with all others reporting that a physician was on call or reachable in case of need. Covering physicians were ER-trained specialists in only one district hospital while four had a generalist with some additional ER-specific training and six had nurses with ER-specific training, representing nine hospitals in total (23 %) Fig. 1.

### Imaging and equipment

**Fig. 2:**
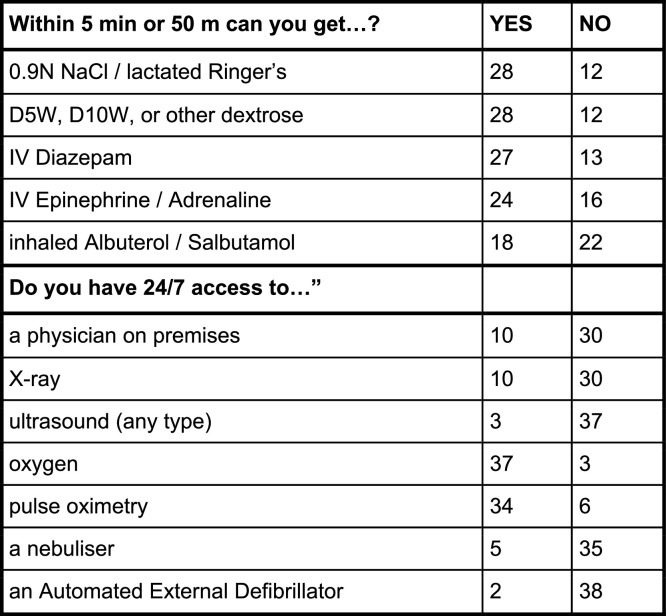
Counts of district hospitals reporting resources.

Ancillary services were similarly distributed. One in four centers boasted 24/7 access to X-rays while only three had continual access to ultrasound. This was in contrast to the near ubiquitous presence of ultrasound machines available at district hospitals. Respiratory support was more common with 37 claiming a continual source of oxygen and 34 able to perform pulse oximetry. Only five respondents reported access to a nebulizer and only two had AEDs available. Fig. 2 summarizes the findings for access within 5 min or a 50 m walk to emergency treatments.

### Medications

Though required by the Ministry of Health quality metrics, IV crystalloids were not close at hand in 30 % of the responding district hospitals. Injectable Diazepam (68 %) was more available than IV Adrenaline (60 %) and less than half (45 %) of respondents could get inhaled Salbutamol within 5 min or a 50 m walk from the bedside.

### Impact of training

**Fig. 3:**
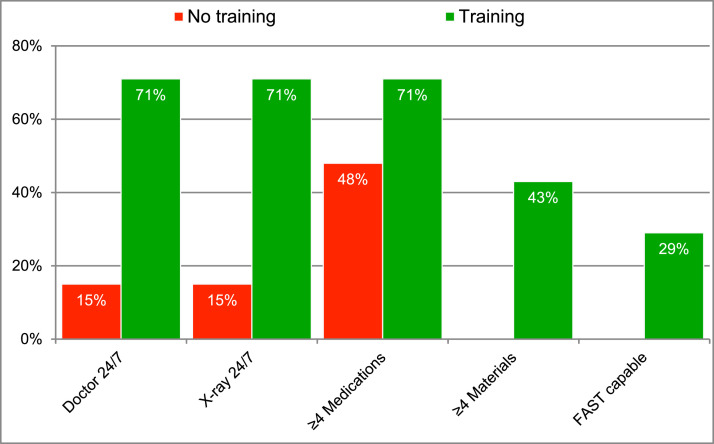
Emergency Department capacity by presence of any trained staff member.

Fig. 3 compares DH with at least one provider trained in emergency medicine (9 respondents) and those without (31 respondents) on the basis of the accessibility of these various resources. Differences were statistically significant (*p*<0.05) for each measure except the presence of at least four of the queried medications and availability of FAST ultrasound. (Comparisons of rural vs urban DH showed no statistically-significant differences in accessibility of these resources.)

**Fig. 4:**
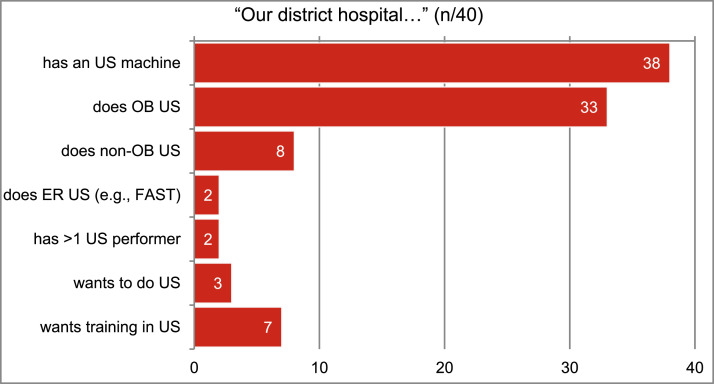
Ultrasound (US) at the district hospital in Burundi.

For ultrasound capacity Fig. 4, summarizes responses from DH in Burundi. Most DH in Burundi (38/40, 95 %) have access to an ultrasound machine though the availability of personnel capable of performing scans drops precipitously when one queries beyond obstetrical US. Emergency POCUS and redundancy of capacity was quite rare (2/40, 5 %).

## Discussion

The objective of this study was to assess the current situation of resources to diagnose and manage critically ill patients in Burundi and to guide efforts to improve the quality of care for the most vulnerable patients.

With the majority of DH reporting and a sample that closely matched the demographics of the country, we consider these results to be representative for Burundi as a whole. While Burundi lags behind some other sub-Saharan African nations in reported physician coverage in the DH, it is challenging to extrapolate norms for the resource-limited context from studies with small samples or that skew urban [34,35]. The vast majority of Burundi's population lives and seeks care outside of the major cities. In these more rural seetings research is often prioritised below direct patient care and metrics reporting to governments and other stakeholders.

### Personnel

Francophone Africa in general and Burundi in particular lack formalised training programs in emergency medicine, helping to explain the relative lack of trained personnel at the DH level. (The authors’ personal familiarity with the staff of regional and national referral hospitals indicates that the situation is no different there.) This may be amenable to change now that the BEC is available in French but trainers are needed before this can be widely disseminated. The preponderance of pilot studies and funded trials through the WHO are in anglophone contexts, possibly due to the preexistence of more established emergency medicine societies and academicians in those countries.

### Material

Only a quarter of DH had X-ray available around the clock, which falls well short of the average for sub-Saharan Africa [5]. The lack of AEDs, similar in Burundi to neighbouring Rwanda [36], may be understandable as other investments such as improved access to blood products are likely higher yield. As non-communicable diseases increase in prevalence, this gap will need to be addressed and cost analyses performed. There can be no doubt that early defibrillation of shockable rhythms improves both cardiac and neurological outcomes. The price of AEDs has come down in the past decade and adhesive patches with extended shelf life more affordable than ever. The absence of AED description or training in the BEC will likely pose an ongoing challenge to widespread adoption of the technology in the LMIC context. Chronic obstructive pulmonary disease and asthma are both significantly prevalent pathologies in the Burundian context where dust in dry season and smoke when clearing fields for planting and from wood-fired indoor cooking pose universal risks to a population that largely cannot afford tobacco products. Allergic reactions and hyperkalemia may be less frequently diagnosed due perhaps to long transport times and lack of laboratory facilities. It is perhaps somewhat reassuring that 18 DH had rapid access to inhaled Salbutamol despite only 5 having nebulisers, implying that inhalers play a significant role in the treatment of these airway-constricting diseases.

### Medications

Perhaps most strikingly, our study showed that only 70 % of DH had crystalloids and only 60 % had IV Adrenaline immediately available to the Emergency Department. These low cost, high impact interventions are already mandated by the government and so are likely to be present at the hospital but apparently not available within a 5 min delay or a 50 m walk. This is perhaps one of the most effective interventions suggested by this study. Simply moving those elements into the space where critically-ill patients are received would likely improve outcomes and can be effected at extremely low cost. A cabinet or tackle box in the Emergency Department with these resuscitation treatments, assuming it could be protected from theft or re-appropriation, could easily solve this problem.

### Training impacts

On the other hand, the vast majority if DH (95 %) had ultrasound devices and most (83 %) were able to do obstetric ultrasound. This bodes well for the future of emergent ultrasound capacity but the technology-training mismatch (only 5 % had the staff trained to perform other emergent ultrasounds) must be addressed. Access to equipment is only one component of the development bundle. Large, fixed US machines may have served well in an era where resolution was poor, studies took 30–60 min, and machines were major capital investments. However, in the third decade of the Twenty-First Century, the technology permits clinicians even in the world's poorest country to access decent quality real-time ultrasound imaging at the bedside. There will be no putting the transistor-based probe back into the POCUS bottle.

Of note, our study showed no significant difference between rural and urban DH in terms of preparedness for providing emergency care, which was part of the planned analysis. The correlation between having at least one staff member with emergency medicine training and having more material resources on hand was significant. It is not difficult to imagine how a local advocate for emergency care or how an administration that values emergency medicine training for the personnel could contribute to greater readiness for critical cases.

### Limitations

Our study had several weaknesses including those of convenience samples and self-reporting. It would be helpful to know what type of training was received and for how long those emergency trained providers have been in place and to evaluate what level of training correlates to improved readiness to render emergency care. Direct inspection of the healthcare facility, as others have done, could help overcome problems associated with recall bias. Access to government reporting and supervision databases could also have furnished a longitudinal view to offset episodic absences of certain elements. Followup studies could also elucidate how that training in obstetrical ultrasound was deployed as a potential model to follow for emergency point of care ultrasound as well as the aforementioned cost-benefit analysis for placing AEDs in district hospitals.

## Conclusion

Burundi needs strategic investment in emergency preparedness and care. Policy initiatives and technology procurements have demonstrated reasonable penetration to the district hospital level, however, trained personnel are essential to develop durable emergency capacity. Stakeholders at the national and international level can support the development of such life-saving diagnostic and therapeutic care with sponsored training for emergency care providers in Burundi and abroad as well as technical advisory support for district hospitals to better utilize existing resources. If you wish to go fast, go alone but if you wish to go far, go together.

## Dissemination of results

Results will be shared with the Ministry of Health (Dr NTIHABOSE Oscar, acknowledged in the article) and were presented via a poster at AfCEM 2022 in Accra, Ghana.

## Authors contribution

Authors contributed as follow to the conception or design of the work; the acquisition, analysis, or interpretation of data for the work; and drafting the work or revising it critically for important intellectual content: TG contributed 58 %, DPB 15 % and CBW 27 %. All authors approved the version to be published and agreed to be accountable for all aspects of the work.

## Declaration of Competing Interest

The authors declare no conflicts of interest.

## Appendix Supplementary materials

Image, application 1
