# Supplementary material for: Essential human and material resources for emergency care in the district hospitals of Burundi
Source: Afr J Emerg Med. 2023 Oct 13;13(4):300–5. doi: 10.1016/j.afjem.2023.09.005 (PMC10582767; doi:10.1016/j.afjem.2023.09.005)
Supplement: Supplementary file 1 [file mmc1.docx]

APPENDIX (data supplement)

Questionnaire : Emergency Preparedness at District Hospitals in Burundi

This survey form seeks to establish the state of resources available to district hospitals in Burundi at the Emergency Department level.

1. Your district hospital name ...................................................…

2. Is there a doctor on duty 24 hours a day, 7 days a week in the Emergency Department?
 - The doctor is available but not present 24/7.
 - Yes, there is a doctor on duty 24/7.
 - No, there is not a doctor on duty 24/7.

 3. Is there at least one staff member who has received specific emergency training?

 - No, no employee has received such training
 - Yes, one or more nurses have received training
 - Yes, one or more physicians have received training
 - Yes, we have an emergency medicine specialist

4. Does the Radiology Department operate 24 hours a day, 7 days a week at your district hospital.
 - Yes, X-rays are available 24/7
 - No, there are times when we cannot perform X-rays

 5. Does the Emergency Department have the equipment listed below available 24 hours a day?

 Yes No
-Oxygen source
-A pulse oximeter
-Nebulizer
-An ultrasound machine

-Automated external defibrillator (AED)

 6. From the patient's bed, do you have to walk more than 50 m or spend more than 5 min to find the medications listed below?

 Yes No
-0.9N NaCl or Ringer’s Lactate
-D5W, D10W or D50W
-IV Adrenaline / Epinephrine
-Albuterol / Salbutamol (spray or nebulizer solution)
-IV Diazepam

 7. For ultrasound, check all that apply

 - Our hospital has an ultrasound machine
 - Our hospital has a clinician who can perform obstetric ultrasound
 - Our hospital has a clinician who can perform non-obstetrical ultrasound (e.g., cardiac echo, abdo ultrasound, etc.)
 - Our hospital has a clinician who performs emergency ultrasounds (e.g., FAST, pneumothorax, etc.)
 - Our hospital has several clinicians who perform all the different ultrasounds
 - Our hospital is looking for a clinician who can do ultrasound
 - Our hospital is looking for ultrasound training for staff
